# “ISA-Lation” of Single-Stranded Positive-Sense RNA Viruses from Non-Infectious Clinical/Animal Samples

**DOI:** 10.1371/journal.pone.0138703

**Published:** 2015-09-25

**Authors:** Fabien Aubry, Antoine Nougairède, Lauriane de Fabritus, Géraldine Piorkowski, Ernest A. Gould, Xavier de Lamballerie

**Affiliations:** 1 Aix Marseille Université, IRD French Institute of Research for Development, EHESP French School of Public Health, EPV UMR_D 190 "Emergence des Pathologies Virales", 13385, Marseille, France; 2 Institut Hospitalo-Universitaire Méditerranée Infection, Marseille, France; Singapore Immunology Network, Agency for Science, Technology and Research (A*STAR), SINGAPORE

## Abstract

Isolation of viral pathogens from clinical and/or animal samples has traditionally relied on either cell cultures or laboratory animal model systems. However, virus viability is notoriously susceptible to adverse conditions that may include inappropriate procedures for sample collection, storage temperature, support media and transportation. Using our recently described ISA method, we have developed a novel procedure to isolate infectious single-stranded positive-sense RNA viruses from clinical or animal samples. This approach, that we have now called "ISA-lation", exploits the capacity of viral cDNA subgenomic fragments to re-assemble and produce infectious viral RNA in susceptible cells. Here, it was successfully used to rescue enterovirus, Chikungunya and Tick-borne encephalitis viruses from a variety of inactivated animal and human samples. ISA-lation represents an effective option to rescue infectious virus from clinical and/or animal samples that may have deteriorated during the collection and storage period, but also potentially overcomes logistic and administrative difficulties generated when complying with current health and safety and biosecurity guidelines associated with shipment of infectious viral material.

## Introduction

Isolation of viruses using cell culture or laboratory animals has, for many years, been the “gold standard” for human and veterinarian clinical virology laboratories [1–2] and also for environmental virology studies [3]. Despite the recent development of sensitive and specific molecular detection methods, in particular in routine virological diagnostic laboratories [4–6], virus isolation remains essential as a non-oriented diagnostic technique and it also provides a reference infectious virus that can be used for phenotypic and genotypic characterisation as well as a variety of applied sciences including antiviral drug-screening assays, structural analysis and studies of the fundamental basis of virus pathogenesis [7].

Conventionally, the isolation of viral pathogens requires infectious virus to be present in the sample. Unfortunately, viability can readily be compromised due to intrinsic fragility of the virus, inappropriate sample collection, unsuitable transportation and/or storage conditions, or when the sample is inactivated prior to shipment or laboratory manipulation, using methods compatible with subsequent analysis techniques (*e*.*g*. heat treatment or the addition of chaotropic reagents for serological or molecular analyses).

Reverse genetics potentially enables the recovery of infectious viruses directly from nucleic acids encoding the viral genomes. The most commonly used “infectious clone” system utilises an engineered DNA copy of the viral genome stably incorporated into a vector and amplified in an appropriate host (most commonly, a plasmid vector in a bacterial host). The use of reverse genetics to recover infectious virus is a particularly attractive option when investigating a large range of field samples previously considered to be intractable for isolation of infectious virus. Indeed, reverse genetics has been used to recover infectious simian immunodeficiency virus and separately adeno-associated virus, neither of which could be recovered directly from clinical samples using conventional tissue culture methods [8–10]. Nevertheless, despite recent improvements in molecular techniques, the protocols remain difficult and laborious [11–12] and are not suitable for routine use in diagnostic laboratories.

We have recently developed a novel, highly sensitive and technically simple method which facilitates recovery of infectious single-stranded positive-sense RNA viruses from nucleic acids. The method, based on the generation of infectious subgenomic amplicons (ISA) involves *(i)* PCR amplification of the complete viral genome as overlapping non-infectious DNA fragments; *(ii)* flanking the first and last fragments respectively in 5’ and 3’ with the human cytomegalovirus promoter (pCMV) and the hepatitis delta ribozyme followed by the simian virus 40 polyadenylation signal (HDR/SV40pA); and *(iii)* transfecting amplicons directly in susceptible cells [13].

Here, we demonstrate that the ISA method is a convenient tool for isolating single-stranded positive-sense RNA viruses from samples that contain inactivated virus. This new procedure, termed "ISA-lation", has now been successfully tested with a variety of single-stranded positive-sense RNA viruses. Based on the results presented herein, the ISA-lation protocol has the potential to rescue infectious viruses from a wide variety of human, animal and environmental field samples that would otherwise have produced negative results under conventional conditions for virus isolation.

## Materials and Methods

### General strategy

**Fig 1:**
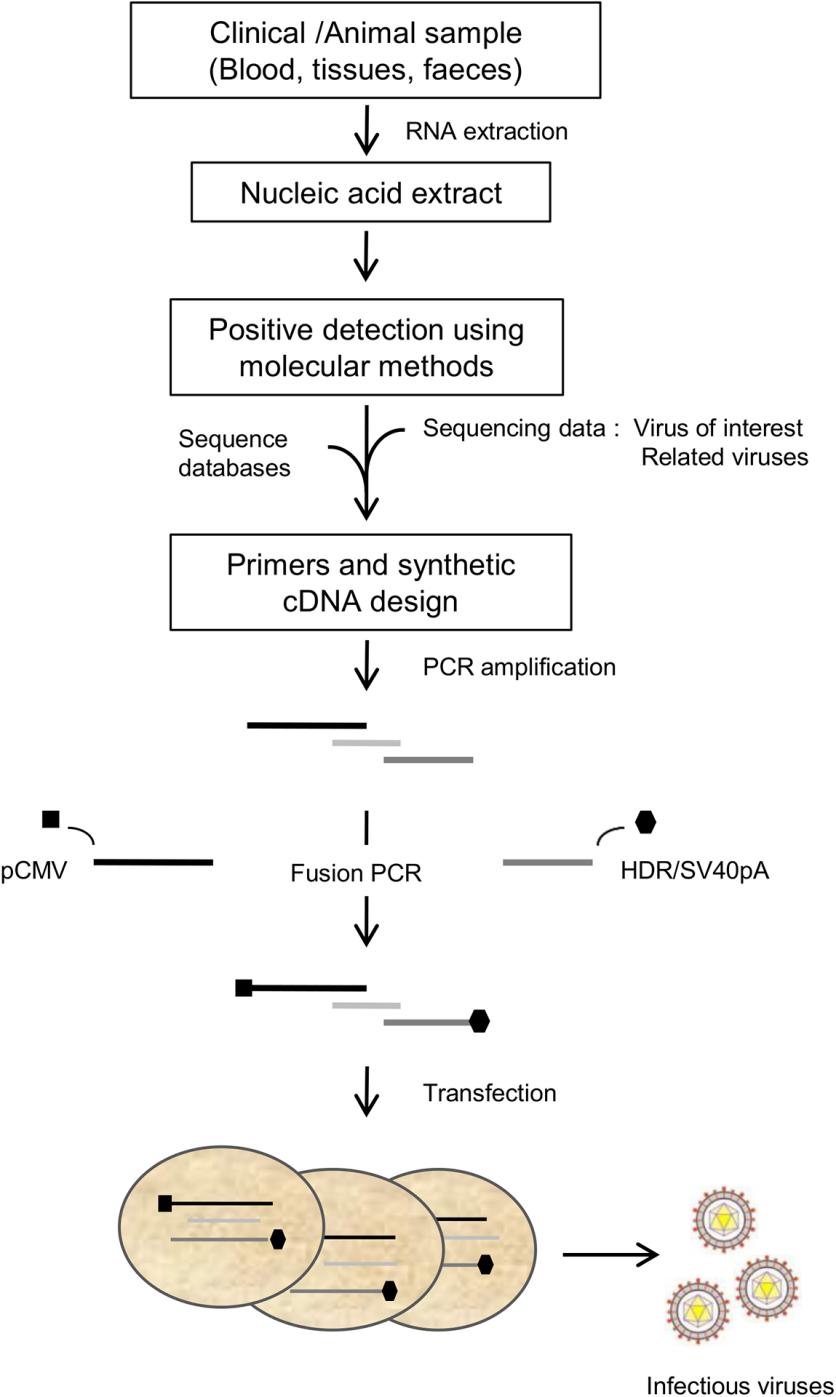
Schematic representation of the ISA-lation method used to recover infectious viruses from clinical samples. Viral RNA directly extracted from clinical samples was used to amplify by RT-PCR the entire viral genome in 3 overlapping cDNA fragments. The human cytomegalovirus promoter (pCMV) and the hepatitis delta ribozyme followed by the simian virus 40 polyadenylation signal (HDR/SV40pA), were then added respectively at the 5’ and 3’ termini of the first and last fragment as described in the Results/Discussion section. Transfection of the 3 final overlapping DNA fragments into permissive cells enabled the isolation and recovery of infectious viruses after 3 to 9 days.

The ISA-lation procedure comprises the following steps (a detailed protocol of each step follows this paragraph) (**Fig 1**):

- Nucleic acid extraction from (inactivated) samples
- Preparation of genomic overlapping PCR fragments
- Cell transfection of amplicons
- Detection of viral production

### Clinical samples used in the current study (Table 1)

Human samples: Because *(i)* biological analysis (virus detection and isolation) were prescribed by physician, *(ii)* only leftovers were used,*(iii)* the results obtained in this study had no impact on patient’s management and *(iv)* oral non-opposition was obtained during hospital admission of the patients, specific consent from patients and ethics committee approval were not required for this study (according to national regulations: “Interprétation des réglementations relatives aux études liées aux DMDIV nécessitant la collecte d’échantillons d’origine humaine; Agence Francaise de Sécurite Sanitaire des Produits de Santé; 30 July 2013”).

**Table 1:** Summary of the different viruses isolated in this study with the ISA-lation method. Nature of the sample used for isolation, cell lines used for transfection and passage, relative quantification of the amount of viral RNA in the sample and after two passages by real-time RT-PCR, infectious titres in supernatant media after two passages by TCID50 assay and presence or absence of cytopathic effect (CPE).

Virus	Nature of the specimen	Viral RNA in the original specimen (copy/mL)	Cell line used for transfection	Cell line used during passages	Viral RNA in supernatant medium after two passages (copy/mL)	TCID_50_/mL in supernatant medium after two passages	Observation of CPE
**TBEV**	Filtrate from mouse brain suspension	2.85 10^5^	HEK-293	BHK-21	1.87 10^7^	3.16 10^6^	Yes
**CHIKV**	Human serum	5.72 10^5^	HEK-293	HEK-293	2.01 10^7^	3.72 10^6^	Yes
**E-30**	Human pharyngeal swab	2.15 10^3^	HEK-293	MRC-5	7.05 10^6^	4.17 10^6^	Yes
	Human stools	1.15 10^6^	HEK-293	MRC-5	5.34 10^7^	3.16 10^6^	Yes

One pharyngeal swab and one stool sample collected during an outbreak in Marseille, France, in 2013, as previously described [14], and testing positive for enterovirus using a one-step RT-PCR method [15]. The enteroviruses were subsequently genotyped as Echovirus 30 (E-30; Human Enterovirus B; genus *Picornavirus*; *i*.*e*. a non-enveloped virus with a single stranded RNA genome of positive polarity, polyadenylated at its 3' end) by direct partial sequencing of the VP1 gene [16].

One human serum testing positive, using a one-step RT-PCR method [17], for the presence of chikungunya virus (CHIKV; genus *Alphavirus*; *i*.*e*. an enveloped virus with a single stranded RNA genome of positive polarity, polyadenylated at its 3' end), collected during the epidemic that is currently affecting the Caribbean [18].

Animal sample: A brain suspension filtrate collected from a five-week-old C57Bl/6J female mouse inoculated intraperitoneally with 2.10^6^ TCID_50_ of the strain Oshima 5–10 of tick-borne encephalitis virus (TBEV; genus *Flavivirus*; an enveloped virus with a single stranded RNA genome of positive polarity, not polyadenylated at its 3' end) and sacrificed 7 days later as previously described [19]. Animal experiments were approved by the Ethics Committee “Marseille-C2EA-14” (ref. #2504) and performed in compliance with French and European regulations (directive 210/63/EU).

### Sample inactivation and extraction of nucleic acids

Samples were inactivated using one volume of AVL buffer (Qiagen, containing guanidinium salts [20]) for one volume of sample. Extraction of nucleic acid was performed using the EZ1 advanced XL machine (Qiagen) with the EZ1 Virus Mini Kit v2.0 (Qiagen).

### Preparation of genomic overlapping amplicons

Three overlapping amplicons were produced from the RNA extract of each clinical sample. RT-PCR assays were performed using the Superscript III One-Step RT-PCR Platinum TaqHifi kit (Life Technologies). The mixture (final volume: 50 μL) contained 25 μL of Reaction Mix, 2 μL of nucleic acid extract, 100 nM of each primer, 1 μL of Enzyme Mix and 20 μL of Nuclease-Free Water. Primers were designed from the full-length sequence of the virus when available (TBEV, CHIKV) or from the alignment of sequences from related viruses retrieved from GenBank (E-30) (**Tables A and B in S1 File**).

The amplicons obtained were as follows (positions according to full-length genomic sequence). TBEV_1: 1–4970, TBEV_2: 3866–8026, TBEV_3: 7851–11100; E-30_1: 6–2287, E-30_2: 2166–5404, E-30_3: 5232–7441; CHIKV_1: 1–3635, CHIKV_2: 3486–7112, CHIKV_3: 6998–11296. Of note, whilst the complete TBEV viral genome was amplified at this stage, this was not the case for the first 6 nucleotides of E-30 virus, and the final 740 and 39 nucleotides at the 3’ end of the CHIKV and E-30 genomes, respectively.

### Addition of the CMV promoter (pCMV) at the 5’ end of the first cDNA fragment

For each virus defined above, the pCMV was amplified by PCR from a previously available plasmid [13] using a forward specific primer and a reverse primer extended with the first ~twenty nucleotides (nt) of the viral genome (TBEV: 20 nt; CHIKV: 25 nt; E-30: 24 nt). This tagged pCMV and the first cDNA fragments were then merged by fusion PCR to obtain the final first DNA genomic fragments (**Table B in S1 File**) (**Fig 1**). All PCR assays were performed using the Platinum PCR SuperMix High Fidelity kit (Life Technologies). The mixture (final volume, 50 μl) consisted of 45 μl SuperMix, 2 μL DNA template at 1 ng/μL and 200 nM of each primer. The final amplicons were obtained as follows (positions according to full-length genomic sequence). TBEV_1: 5’ terminus of the pCMV-4054, CHIKV_1: 5’ terminus of the pCMV-3607.

### Addition of the hepatitis delta ribozyme followed by the simian virus 40 polyadenylation signal (HDR/SV40pA) at the 3’ end of the last cDNA fragment

In the case of TBEV (no 3’-polyadenylated genome), the HDR/SV40pA was amplified by PCR from a previously available plasmid [13] using a forward primer extended with the last 21 nucleotides of the viral genome. This tagged HDR/SV40pA and the third cDNA fragment were then merged by fusion PCR (**Table B in S1 File**) (**Fig 1**).

For RNA viruses with a 3’-polyadenylated genome (*i*.*e*., CHIKV and E-30), the 740 (CHIKV) or 39 (E-30) final nucleotides of the viral genome, the polyA tail and the HDR/SV40pA were synthesized *de novo* (Genscript) (**Notes A and B in S1 File**) and then merged by fusion PCR with the pre-amplified final third of the viral genome (**Table B in S1 File**) (**Fig 1**).

The final amplicons were obtained as follows (positions according to full-length genomic sequence). TBEV_3: 7935–3’ terminus of the HDR/SV40pA, CHIKV_3: 7023–3’ terminus of the HDR/SV40pA.

### Cell preparation and transfection

All cells were cultured at 37°C with 5% CO_2_ using reagents from Life Technologies. Baby Hamster Kidney (BHK-21) cells: Minimal Essential Medium with 7% heat-inactivated foetal bovine serum and 1% Penicillin/Streptomycin (PS); Human Embryonic Kidney 293 (HEK-293): same medium supplemented with 1% of non-essential amino acids; Human foetal lung fibroblast (MRC-5): Basal Medium Eagle with 10% FBS and 1% PS.

For a given virus, 1 μg of an equimolar mix of the 3 subgenomic amplicons was mixed with 12 μl of Lipofectamine 2000 (Life Technologies) in 600 μl of Opti-MEM medium (Life Technologies) and added to a 12.5 cm^2^ culture flask containing sub-confluent HEK 293 cells in 1 mL of medium without antibiotics. After a 4 hour incubation period, the supernatant medium was removed, cells were washed twice (HBSS; Life Technologies) and 3 mL of fresh medium were added.

### Detection of viral production

The supernatant medium was harvested when gross cytopathic effect (CPE) was observed (4–8 days depending on the cell type and the rate of virus replication), clarified by centrifugation, and then sequentially passaged twice using the same cell type for the CHIKV, BHK-21 cells for TBEV and MRC-5 cells for E-30 virus. These sequential passages were performed to ensure the complete elimination of the original DNA used during the transfection step. Sequential passages were performed by inoculating 333 μL of clarified infectious supernatant medium onto cells in a 12.5 cm^2^ culture flask containing 666 μL of medium. After incubation for 2 hours, cells were washed twice (HBSS) and 3 mL of fresh medium was added. The supernatant medium was harvested after 2–6 days, clarified by centrifugation, aliquoted and stored at -80°C. Clarified supernatant media (virus stocks) were used to performquantification of viral RNA, TCID_50_ assay, and whole-genome sequencing.

#### Real time PCR and RT-PCR assays

Nucleic acids were extracted from supernatant media using the EZ1 mini virus 2.0 kit and the EZ1 advanced XL machine (both from Qiagen) according to the manufacturer's instructions.

To assess the production of infectious viruses and to ensure that positive detection was not the result of DNA contamination, viral RNA and DNA sequences were semi-quantified using qRT-PCR (GoTaq® qPCR Master Mix kit, Promega) and qPCR (Takyon No Rox Probe MasterMix kit, Eurogentec) assays under standard amplification conditions at a 60°C hybridisation temperature.

For qRT-PCR tests, the mixture (final volume: 20 μL) contained 10 μL of Master Mix 2X Reaction Buffer, 0.5 μM of each primer, 0.3 μL of probe, 0.5 μL of GoSCRIPT RT Mix, 0.87 μL of Nuclease-Free Water and 7.5 μL of extracted nucleic acids. For qPCR tests, the mixture (final volume: 25 μL) contained 12.5 μL of TakyonTM MasterMix, 0.5 μM of each primer, 0.3 μL of probe, 3.55 μL of Nuclease-Free Water and 7.5 μL of extracted nucleic acids (**Table C in S1 File**).

The quantity of viral RNA (number of genome copies per mL) was calculated by comparison with calibrated standard curves produced from serial dilutions of nucleic acids.

#### Tissue Culture Infectious Dose 50 (TCID_50_) assay

For each determination, a 96-well plate culture containing either 20,000 human adrenal carcinoma (SW13) cells (for TBEV), MRC-5 cells (for E-30) or African green monkey kidney (Vero) cells (for CHIKV) in 100 μL of medium per well was inoculated with 50 μL of serial 10-fold dilutions of clarified infectious supernatant medium. Each row included 6 wells of the same dilution and two negative controls. The plates were incubated for 7 days and read for absence or presence of CPE in each well. The determination of the TCID_50_/mL was performed using the method of Reed and Muench [21].

#### Sequence analysis of full-length genomes

Next-Generation Sequencing (NGS) of complete genomes was performed using the Ion PGM Sequencer [22] (Life Technologies) and analyses were conducted with the CLC Genomics Workbench 6 software. RNA was extracted from supernatant medium using the EZ1 mini virus 2.0 kit and the EZ1 advanced XL machine (both from Qiagen) according to the manufacturer's instructions. Complete viral genomes were amplified in three or four fragments using specific sets of primers with the Superscript III One-Step RT-PCR Platinum TaqHifi kit (Life Technologies) as described above. Amplified DNA was analysed using the Ion PGM Sequencer according to the manufacturer's instructions. The read sequences obtained were trimmed, first using quality score, then by removing the primers used to amplify the complete genome by RT-PCR and finally at the 5’ and 3’ termini by systematically removing 6 nt. Only reads with a length greater than 29 nt were used and mapped for comparison to thesequences of the corresponding viruses: CHIKV H20235/STMARTIN/2013 strain (available through the European Virus Archive website under reference no. 1540), the TBEV Oshima 5–10 strain (GenBank accession number AB062063) or the E-30 1-MRS2013 strain (GenBank accession number KF920598). Mutation frequencies (proportion of viral genomes with the mutation) for each position were calculated simply as the number of reads with a mutation compared to the reference divided by the total number of reads at that site. All reads are available in NCBI Sequence Read Archive; BioProject PRJNA291127; accession number SRP062989.

For viruses (CHIKV and TBEV) for which the sequence of the original virus contain in the sample was known, the integrity of the sequenced genomes was considered as verified when the nucleotides similarity was ≥99.9%.

For viruses (E-30) for which the sequence of the original virus contain in the sample was unknown, the integrity of the sequenced genomes was verified by aligning with the closest complete genomes available in the database.

## Results

We attempted to recover infectious single-stranded positive-sense RNA viruses from inactivated biological specimens (pharyngeal swab, stools, serum and brain tissue) using a method derived from the previously described ISA method [13]. For each specimen, the procedure was designed to produce an equimolar mix of 3 overlapping DNA fragments covering the complete viral genome with pCMV and HDR/SV40pA sequences at the 5'and 3' termini, respectively (**Fig 1**), and to transfect HEK-293 cells with this preparation.

The amount of TBEV RNA in the brain suspension filtrate was 2.85 10^5^ copies/mL. Following transfection, a cytopathic effect (CPE) was observed at day 8. Supernatant medium was subsequently sequentially passaged twice on BHK-21 cells. CPE was observed at each passage. On completion of the second passage (P2), the quantities of viral RNA measured in the culture supernatant medium were 1.87 10^7^ copies/mL (*i*.*e*., higher than in the initial sample) and the titre of infectious particles was 3.16 10^6^ TCID_50_/mL (**Table 1**). NGS complete genomic sequencing confirmed the integrity of the genome structure and the genetic similarity with the TBEV Oshima 5–10 strain used for infecting mice (**Tables D and E in S1 File**).

The amount of CHIKV RNA in the original serum was 5.72 10^5^ copies/mL. Following transfection, CPE was observed at day 8. Supernatant medium was then sequentially passaged twice on HEK-293 cells. CPE was observed at each passage. At passage two, the amounts of viral RNA and infectious particles in the culture supernatant medium were respectively 2.01 10^7^ copies/mL (*i*.*e*., higher than in the initial sample) and 3.72 10^6^ TCID_50_/mL (**Table 1**). NGS complete genomic sequencing confirmed the integrity of the genome structure and the genetic similarity with CHIKV H20235/STMARTIN/2013 strain, isolated during the same epidemic [18] (**Tables D and E in S1 File**).

The amount of E-30 RNA in the original samples was 2.15 10^3^ copies/mL in the pharyngeal swabs and 1.15 10^6^ copy/mL in stools. CPE was observed at day 8 post transfection. Supernatant medium was sequentially passaged twice onto MRC-5 cells, with renewed CPE. The amounts of viral RNA and infectious particles in the P2 supernatant media deriving from the pharyngeal swab were 7.05 10^6^ copies/mL (*i*.*e*., higher than in the initial sample) and 4.17 10^4^ TCID_50_/mL. They were 5.34 10^7^ copies/mL (*i*.*e*., higher than in the initial sample) and 3.16 10^4^ TCID_50_/mL in supernatant media derived from stools (**Table 1**). NGS complete genomic sequencing confirmed the integrity of the genome structure and the genetic similarity with the E-30 1-MRS2013 strain isolated during the same outbreak [14] (**Tables D and E in S1 File**).

## Discussion

We have developed a technically simple but scientifically sophisticated procedure designated “ISA-lation” which facilitates the rescue of infectious single-stranded positive-sense RNA viruses from clinical samples in which, for a variety of reasons, virus infectivity has been lost but genomic RNA has retained sufficient fidelity to enable authentic RT-PCR amplification. Here, we have validated this rescue procedure for viruses belonging to three different families which harbour different characteristics (enveloped; non-enveloped; polyadenylated; non-polyadenylated), starting from a variety of inactivated clinical samples (serum, brain tissue, pharyngeal swabs and stools).

This procedure possesses several important advantages over previous molecular identification methods. Moreover, ISA-lation overcomes problems associated with health, safety and security as follows. Firstly, ISA-lation provides a method of rescuing viruses from clinical samples or cell cultures that no longer contain detectable infectious viruses resulting from inappropriate sample collection, transportation and/or inappropriate storage conditions. Secondly, and importantly, national and international health, safety and security regulations applicable to development, exchange and transportation of infectious materials, impose increasing costs and preparation times and highly significant restriction on the shipment of infectious agents. Exploitation of the ISA-lation protocol provides biological tools for fundamental research, virus discovery and rescue in a diagnostic context and incidentally circumvent most, if not all, of the problems associated with safe shipment of high level pathogens. Thus, clinical samples suspected of containing pathogenic viruses that prove to be unretrievable by conventional virus isolation procedures are now potentially amenable to the ISA-lation rescue process via technically simple procedures and at relatively low cost. Furthermore, by deliberately inactivating samples directly after collection, this procedure effectively reduces the perceived risk of infection and also preserves the viral RNA for molecular diagnosis and subsequent virus recovery using the ISA-lation procedure. An additional advantage is that field sampling procedures are significantly simplified in the absence of the constraints associated with the need for preserving virus infectivity. Finally, the ISA-lation procedure also enables recovery of a particular virus in the presence of interfering agents such as multi-resistant bacteria, fungi or even other viruses that may replicate faster than the targeted virus population. Furthermore, in contrast with some reverse genetics protocols that include cloning steps, ISA-lation, which is based on production of cDNA fragments using PCR, could conserve the genetic diversity of the viral population present in the original specimen [23].

Thus, the ISA-lation procedure for rescuing infectious viruses from clinical/animalsamples will complement other molecular technologies that include real time RT-PCR which can be used to detect and quantify viral genomes in targeted samples, Next-Generation Sequencing methods that enable genomic characterisation directly from the sample, and therefore facilitate the design of ISA primers [18], and *de novo* DNA synthesis technologies, which could be alternatively used to generate cDNA fragments [13].

In conclusion, the procedure that we have termed ISA-lation represents a specific, sensitive, novel and technically simple procedure for rescuing positive-stranded RNA genomes as infectious viruses from clinical and/or animalsamples that would otherwise fail to yield infectious virus if conventional virus isolation procedures were used. For example, many samples tested in diagnostic laboratories may have deteriorated due to poor collection and storage methods. Alternatively, the infectious virus may be undetectable because it is present below the threshold level for detection by conventional methods, as for example in cases of persistent infection. In other cases, the sample may have been tested and found positive by molecular biological methods but failed to yield infectious virus using cell culture isolation methods. Using the much more sensitive ISA-lation procedure, the only essential requirement is that the sample must contain amplifiable full-length genomic RNA. In addition, the ISA-lation procedure has a major practical application which potentially overcomes logistic and administrative difficulties generated when complying with current health and safety and biosecurity guidelines. This applies when working with hazardous virus pathogens. Using ISA-lation procedures, a hazardous positive-stranded RNA virus can be safely transported as separate DNA fragments from one compliant laboratory to another without the need for expensive safety containment procedures. Clearly, the next step is to develop the ISA-lation method for other viruses.

## Supporting Information

S1 File Note A. Sequence of the synthetic cDNA fragment used for the E-30. Note B. Sequence of the synthetic cDNA fragment used for the CHIKV. Table A. External primers used for the preliminary amplifications of the different elements composing the first and the last fragment. Table B. Primers used to obtain cDNA fragments used for transfection. Table C. Primers and probes used for the Real time PCR and RT-PCR assays. Table D. Mutations detected by NGS in the complete genomes of the ISA-lated viruses. Table E. Characteristics of the mutations detected by NGS in the complete genomes of the ISA-lated viruses.
(DOCX)
