# Supplementary material for: “ISA-Lation” of Single-Stranded Positive-Sense RNA Viruses from Non-Infectious Clinical/Animal Samples
Source: PLoS One. 2015 Sep 25;10(9):e0138703. doi: 10.1371/journal.pone.0138703 (PMC4583506; doi:10.1371/journal.pone.0138703)
Supplement: S1 File — Note B. Sequence of the synthetic cDNA fragment used for the CHIKV. Table A. External primers used for the preliminary amplifications of the different elements composing the first and the last fragment. Table B. Primers used to obtain cDNA fragments used for transfection. Table C. Primers and probes used for the Real time PCR and RT-PCR assays. Table D. Mutations detected by NGS in the complete genomes of the ISA-lated viruses. Table E. Characteristics of the mutations detected by NGS in the complete genomes of the ISA-lated viruses. (DOCX) [file pone.0138703.s001.docx]

**S1 File: Supporting Information**

“ISA-lation” of [Single-Stranded Positive-Sense RNA Viruses](http://www.ncbi.nlm.nih.gov/pubmed/25053561) from Non-Infectious Clinical/Animal Samples

Fabien Aubry^1*^, Antoine Nougairède^1-2^, Lauriane de Fabritus^1^, Géraldine Piorkowski^1^, Ernest A. Gould^1^, Xavier de Lamballerie^1-2^

1. Aix Marseille Université, IRD French Institute of Research for Development, EHESP French School of Public Health, EPV UMR_D 190 "Emergence des Pathologies Virales", 13385, Marseille, France.
2. Institut Hospitalo-Universitaire Méditerranée Infection, Marseille, France.

* Corresponding author

E-mail: [fab1.aubry@gmail.com](mailto:fab1.aubry@gmail.com)

**Table of Contents**

**Supporting Notes**

**Note A.** Sequence of the synthetic cDNA fragment used for the E-30

**Note B.** Sequence of the synthetic cDNA fragment used for the CHIKV

**Supporting Tables**

**Table A.** External primers used for the preliminary amplifications of the different elements composing the first and the last fragment

**Table B.** Primers used to obtain cDNA fragments used for transfection

**Table C.** Primers and probes used for the Real time PCR and RT-PCR assays

**Table D.** Global analysis of the mutations detected by NGS in the complete genomes of

the ISA-lated viruses

**Table E.** Characteristics of the mutations detected by NGS in the complete genomes of

the ISA-lated viruses.

**Supporting Notes**

**Note A. Sequence of the synthetic cDNA fragment used for the E-30**

This fragment contains the last 39 nucleotides (underlined) of the viral genome, the polyA tail and the HDR/SV40pA (highlighted in grey).

AACGGTGTGGTAGGGGTAAATTCTCCGCATTCGGTGCGGAAAAAAAAAAAAAAAAAAAAAAAAAAGGCCGGCATGGTCCCAGCCTCCTCGCTGGCGCCGGCTGGGCAACATTCCGAGGGGACCGTCCCCTCGGTAATGGCGAATGGGACTCGCGACAGACATGATAAGATACATTGATGAGTTTGGACAAACCACAACTAGAATGCAGTGAAAAAAATGCTTTATTTGTGAAATTAAGCGCTGGCATTGACCCTGAGGTTTACCCTCACAACGTTCCAGTATACGTGGCCGGCCACGTGCGGCCGC

**Note B. Sequence of the synthetic cDNA fragment used for the CHIKV**

This fragment contains the last 740 nucleotides of the viral genome (sequence underlined), the polyA tail and the HDR/SV40pA (highlighted in grey).

GTGGTGCTATGCGTGTCGTTTAGCAGGCACTAACTTGACAACTAGGTATGAAGGCATACGCGTCCCTAAAGAGACACACCGCATATAGCTAGGAATCAACAGATAAGTATAGATCTAAGGGCTGAACAACCCCTGAATAACAAAATATAAAAATCAACAAAAATCATAAAATAGAAAACTAGAAATAGAAGTAGGTAAGAAGGTATATGTGTCCCCTAAGAGACACACCATATATAGCTAAGAATCAATAGATAAGCATAGATCAAAGGGCTGAACAACCCCTGAATAATAACAAAATATAAAAACCAATAAAAATCATAAAATAGAAAACCACAAATAGAAGTAGTTCAAAGGGCTATAAAACCCCTGAATAGTAACAAAATATAAAACTAATAAAAATCAAACGAATACCATAATTGGCAATCGGAAGAGATGTAGGTACTTAAGCTTCTTAAAAGCAGCCGAACTCGCTTTGAGATGTAGGCGTAGCACACCGAACTCTTCCACGATTCTCCGAACCCACAGGGACGTAGGAGATGTTCAAAGTGACTATAAAACCCTGAACAGTAATAAAATATAAAATTAATAATGAGTACCATAATTGGCAAATGGAAGAGACGTAGGTACTAAGCTTCTTAAAAGCAGCCGAACTCACTTTGAGATGTAGGCATAGCATACCGAACTCTTCCACGATTCTCCGAACCCATAGGGACGTAGGAGATGTTATTTTGTTTTTAATATTTCAAAAAAAAAAAAAAAAAAAAAAAAAAAAAAAAAAAAAAAAGGCCGGCATGGTCCCAGCCTCCTCGCTGGCGCCGGCTGGGCAACATTCCGAGGGGACCGTCCCCTCGGTAATGGCGAATGGGACTCGCGACAGACATGATAAGATACATTGATGAGTTTGGACAAACCACAACTAGAATGCAGTGAAAAAAATGCTTTATTTGTGAAATTAAGCGCTGGCATTGACCCTGAGGTTTACCCTCACAACGTTCCAGTATACGTGGCCGGCCACGTGCGGCCGC

**Supporting Tables**

**Table A.** External primers used for the preliminary amplifications of the different elements composing the first (pCMV and the beginning of the viral genome) and the last fragment (end of the viral genome and the HDR/SV40pA). Primers located respectively at the 5’ and 3’ terminus of the pCMV (¶) and the HDR/SV40pA (£).

| **Virus** | **Component** | **Position** | **Length** | **Primer Forward** | **Primer Reverse** |
| --- | --- | --- | --- | --- | --- |
| TBEV | pCMV | ¶ - 20 | 801 | GAATAAGGGCGACACGGAAATGT | ATGCACGTGCAAGAAAATCTCGGTTCACTAAACGAGCTCT |
| TBEV | Fragment I | 1- 4970 | 4970 | AGATTTTCTTGCACGTGCAT | CCCACCTGTGTCCAGGAGC |
| TBEV | HDR/SV40pA | 11060- £ | 237 | TGACTCGGAAAAACACCCGCTGGCCGGCATGGTCCCAGC | TACTGGAACGTTGTGAGGGTAAAC |
| TBEV | Fragment III | 7851-11100 | 3249 | GCCTGGCTTGAGGAACGAGGTTACG | AGCGGGTGTTTTTCCGAGTC |
| CHIKV | pCMV | ¶ - 25 | 806 | GAATAAGGGCGACACGGAAATGT | GGCTACGTGTGTCTCACGCAGCCATCGGTTCACTAAACGAGCTCT |
| CHIKV | Fragment I | 1-3635 | 3635 | ATGGCTGCGTGAGACACAC | GTCCACGATAGTCAATTTGCAGTTGT |
| CHIKV | 3’end of the viral genome-HDR/SV40pA | 11269- £ | 1000 | GTGGTGCTATGCGTGTCGT | TACTGGAACGTTGTGAGGGTAAAC |
| CHIKV | Fragment III | 6998-11296 | 4299 | GGGTGTTGGAAGATCGTCTGACA | GCCTGCTAAACGACACGCAT |
| E-30 | pCMV | ¶ - 28 | 809 | GAATAAGGGCGACACGGAAATGT | GGTGGGAGCAACCCACAGGCTGTTTTAACGGTTCACTAAACGAGCTCT |
| E-30 | Fragment I | 6-2287 | 2282 | AACAGCCTGTGGGTTGCTCCCA | GTGTATGCGTCGCTGGTAACA |
| E-30 | 5’end of the viral genome-HDR/SV40pA | 7409- £ | 281 | AACGGTGTGGTAGGGGTAAA | TACTGGAACGTTGTGAGGGTAAAC |
| E-30 | Fragment III | 5232-7441 | 2210 | GCAGAGCATTCATCTGTCTGCA | CGAATGCGGAGAATTTACCCCT |

**Table B. Primers used to obtain cDNA fragments used for transfection**

I, II and III designate respectively the first, the second and the third cDNA fragments obtained by PCR. bp: base pair. Primers located respectively at the 5’ and 3’ terminus of the pCMV (¶) and the HDR/SV40pA (£).

| **Virus** | **cDNA Fragment** | | **Primer Forward** | **Position** | **Primer Reverse** | **Position** |
| --- | --- | --- | --- | --- | --- | --- |
|  | **Number** | **Length** |  |  |  |  |
| TBEV | I | 4812 bp | CACCCAACTGATCTTCAGCATCT | ¶ | GCCACGCCCAGGAAGAGCATGA | 4033-4054 |
|  | II | 4160 bp | CTGGGATTGCCAAGCGAGG | 3866-3885 | CAACCCAGGCTTGTCACCATCTTT | 8003-8026 |
|  | III | 3338 bp | GCAGCTTCTCGACCGGCTGTCATC | 7935-7959 | CTCAGGGTCAATGCCAGCGCTT | £ |
| CHIKV | I | 4393 bp | CACCCAACTGATCTTCAGCATCT | ¶ | GTTTAACCCACCCTCTGAAGCA | 3586-3607 |
|  | II | 3626 bp | TACGAATGAGTACAACATGCCGAT | 3486-3509 | ATCCAAGTAGCGCATCTGGCT | 7092-7112 |
|  | III | 5221 bp | ATCTGCATGCGCGGCCTTCAT | 7023-7043 | CTCAGGGTCAATGCCAGCGCTT | £ |
| E30 | I | 3036 bp | CACCCAACTGATCTTCAGCATCT | ¶ | TCGCTGGTAACATATCTGTAGT | 2256-2277 |
|  | II | 3239 bp | GGAAAGATGCCATGCTCGGGA | 2166-2186 | TTCAAATGCTGGACCTTGCACT | 5383-5404 |
|  | III | 2394 bp | ATCAGTGGCTGGCATAATATACA | 5272-5294 | CTCAGGGTCAATGCCAGCGCTT | £ |

**Table C. Primers and probes used for the Real time PCR and RT-PCR assays**

| **Virus** | **Position** | **Length** | **Primer Forward** | **Probe** | **Primer Reverse** |
| --- | --- | --- | --- | --- | --- |
| TBEV | 10236-10338 | 102 | GCAGAGTGGGCCAGGAACAT | TCGGACAAGAGAAGTTCAAGGACT | TCCTGCATGGATCGGCATGAC |
| CHIKV | 2631-2810 | 179 | TGACCGCCATTGTGTCATCGTTG | CTGGAGACCTCGTGTTAACGTGCTTCAG | GACCTCGTATCCACGATAGTCA |
| E-30 | 456-601 | 145 | CCCCTGAATGCGGCTAATCC | GGACACCCAAAGTAGTCGGTTCC | ATTGTCACCATAAGCAGCCA |

**Table D. Global analysis of the mutations detected by NGS in the complete genomes of the ISA-lated viruses.** The nature, localisation (synonymous (S) or non synonymous (NS) when present in coding regions or UnTranslated Region (UTR)) and frequency (proportion of viral genomes with the mutation; divided into two categories: > 80% or between 10 and 80%) are detailed.

| **Virus** | | **Mutations** | | | | | |
| --- | --- | --- | --- | --- | --- | --- | --- |
|  |  | **Fixed mutations (frequency >80%)** | | | **Other mutations (Frequency 10-80%)** | | |
|  |  | **S** | **NS** | **UTR** | **S** | **NS** | **UTR** |
| **TBEV** | | 0 | 1 | 0 | 3 | 5 | 1 |
| **CHIKV** | | 5 | 3 | 1 | 0 | 0 | 0 |
| **E-30** | **Human pharyngeal swab** | 158 | 14 | 13 | 2 | 8 | 0 |
|  | **Human stools** | 26 | 6 | 3 | 3 | 4 | 0 |

**Table E. Characteristics of the mutations detected by NGS in the complete genomes of the ISA-lated viruses.** Characteristics of the mutations are described for each mutation: position on the genome, mutation compared with the sequence of reference, mutation frequency and the coverage at each position.

| TBEV | | | | |
| --- | --- | --- | --- | --- |
| Position | Sequence of reference | Mutation | Mutation Frequency | Coverage |
| 481 | C | T | 0,527949327 | 6315 |
| 1529 | C | T | 0,361190612 | 6988 |
| 1566 | A | G | 0,358887581 | 6797 |
| 2189 | C | A | 0,228914348 | 9165 |
| 2196 | G | T | 0,235793824 | 8712 |
| 3515 | A | G | 0,526947705 | 3749 |
| 3879 | A | G | 0,530398833 | 4112 |
| 4654 | A | G | 1 | 4097 |
| 6475 | T | C | 0,997889248 | 8055 |
| 10802 | G | A | 0,516161114 | 2014 |

| CHIKV | | | | |
| --- | --- | --- | --- | --- |
| Position | Sequence of reference | Mutation | Mutation Frequency | Coverage |
| 880 | A | C | 0,996810773 | 2840 |
| 1389 | T | C | 0,991687448 | 2408 |
| 1425 | T | A | 0,994224422 | 2425 |
| 1540 | T | C | 0,998660714 | 2249 |
| 2041 | A | G | 0,994535519 | 2749 |
| 2676 | A | G | 0,993995055 | 2831 |
| 4108 | A | G | 0,985507246 | 2277 |
| 6610 | T | C | 0,994587843 | 2411 |

| E30 Human stools | | | | |
| --- | --- | --- | --- | --- |
| Position | Sequence of reference | Mutation | Mutation Frequency | Coverage |
| 690 | T | A | 0,998665777 | 1504 |
| 699 | A | G | 0,980900409 | 1467 |
| 760 | A | T | 0,986873508 | 838 |
| 838 | C | T | 0,998756219 | 804 |
| 943 | T | C | 0,988408851 | 950 |
| 1105 | A | G | 0,995717345 | 934 |
| 1369 | T | A | 0,99863388 | 733 |
| 1388 | A | G | 0,911170929 | 743 |
| 1786 | T | C | 0,990610329 | 640 |
| 1882 | T | C | 1 | 565 |
| 2098 | T | C | 0,995951417 | 495 |
| 2146 | G | A | 0,561173533 | 1602 |
| 2280 | C | T | 0,167598566 | 6982 |
| 2352 | C | T | 0,157027103 | 7179 |
| 2401 | A | G | 0,72897402 | 4552 |
| 2419 | G | A | 0,993165731 | 4104 |
| 2442 | A | T | 0,553558237 | 4068 |
| 2674 | T | C | 0,995041322 | 605 |
| 2730 | A | G | 0,104529617 | 576 |
| 2917 | C | T | 0,998379254 | 617 |
| 3160 | C | T | 0,998556999 | 693 |
| 3455 | T | A | 0,993235626 | 887 |
| 3476 | A | G | 0,120231214 | 865 |
| 3808 | C | T | 0,967391304 | 184 |
| 3920 | A | G | 1 | 212 |
| 4450 | T | A | 1 | 176 |
| 4490 | T | C | 0,994219653 | 173 |
| 4945 | T | C | 0,969543147 | 197 |
| 4985 | C | T | 1 | 176 |
| 5093 | G | A | 0,93452381 | 168 |
| 5155 | T | C | 0,986547085 | 227 |
| 5626 | T | C | 0,995983936 | 997 |
| 5665 | A | G | 0,999077491 | 1086 |
| 5722 | T | C | 0,997379913 | 1145 |
| 5842 | C | T | 0,997398092 | 1157 |
| 6568 | C | A | 0,97649919 | 1238 |
| 6634 | C | T | 0,995854063 | 1209 |
| 6760 | C | T | 0,99905838 | 1074 |
| 6817 | C | T | 0,997201493 | 1072 |
| 6886 | A | G | 0,980909091 | 1100 |
| 6904 | T | C | 0,804922516 | 1097 |
| 7023 | G | A | 0,991116751 | 788 |

| E30 Human pharyngeal swab | | | | |
| --- | --- | --- | --- | --- |
| Position | Sequence of reference | Mutation | Mutation Frequency | Coverage |
| 87 | T | A | 0,984494563 | 4966 |
| 90 | T | C | 0,995665635 | 4904 |
| 97 | T | C | 0,983695652 | 4968 |
| 117 | C | T | 0,998538279 | 5480 |
| 242 | A | G | 0,99549714 | 8220 |
| 499 | A | T | 0,987322893 | 10728 |
| 662 | T | C | 0,882047812 | 8868 |
| 690 | T | A | 0,996114619 | 8241 |
| 699 | A | T | 0,984003281 | 7314 |
| 720 | G | A | 0,994571429 | 7007 |
| 757 | A | G | 0,996511259 | 3160 |
| 787 | A | C | 0,99198461 | 3125 |
| 823 | T | C | 0,995483871 | 3100 |
| 856 | T | A | 0,999048827 | 3163 |
| 880 | C | T | 0,995155919 | 3321 |
| 940 | C | T | 0,991455426 | 3511 |
| 1108 | G | A | 0,99755919 | 4099 |
| 1144 | T | C | 0,999286054 | 4221 |
| 1153 | G | A | 0,991559203 | 4266 |
| 1195 | T | C | 0,996773326 | 3719 |
| 1198 | G | A | 0,986490138 | 3701 |
| 1219 | A | G | 0,992669862 | 3574 |
| 1313 | C | T | 0,995171998 | 3314 |
| 1336 | A | G | 0,991591405 | 3211 |
| 1385 | C | T | 0,996673597 | 2407 |
| 1399 | A | C | 0,997914929 | 2398 |
| 1426 | A | G | 0,994953743 | 2378 |
| 1453 | T | C | 0,996617336 | 2366 |
| 1465 | C | T | 0,996107266 | 2313 |
| 1531 | C | T | 0,987901544 | 2397 |
| 1534 | T | C | 0,991752577 | 2425 |
| 1600 | C | T | 0,995028998 | 2415 |
| 1651 | A | G | 0,990886495 | 2414 |
| 1663 | C | T | 0,991779696 | 2434 |
| 1687 | A | T | 0,992969396 | 2420 |
| 1712 | C | T | 0,997900042 | 2382 |
| 1786 | T | C | 0,986074848 | 2299 |
| 1849 | G | A | 0,991765782 | 2186 |
| 1861 | A | G | 0,99543379 | 2195 |
| 1905 | C | T | 0,754040404 | 1980 |
| 1921 | T | C | 0,995769434 | 1891 |
| 2002 | C | T | 0,998184019 | 1653 |
| 2023 | C | T | 0,995723885 | 1637 |
| 2047 | T | C | 0,994890138 | 1960 |
| 2104 | A | G | 0,994818653 | 1931 |
| 2146 | G | A | 0,320669056 | 8370 |
| 2152 | C | T | 0,99471707 | 8518 |
| 2236 | A | G | 0,981217078 | 14438 |
| 2299 | T | C | 0,996447448 | 23082 |
| 2326 | T | C | 0,997766899 | 23287 |
| 2335 | G | A | 0,965653153 | 23090 |
| 2380 | G | T | 0,998344636 | 18732 |
| 2407 | A | G | 0,997094525 | 15490 |
| 2419 | G | A | 0,996795501 | 15301 |
| 2455 | C | T | 0,989035383 | 14866 |
| 2467 | C | T | 0,984394251 | 2440 |
| 2488 | A | G | 0,99958489 | 2410 |
| 2509 | A | G | 0,985749186 | 2458 |
| 2515 | C | T | 0,996407186 | 2510 |
| 2575 | G | A | 0,996517028 | 2586 |
| 2584 | C | T | 0,996521067 | 2587 |
| 2599 | G | A | 0,98649722 | 2518 |
| 2605 | G | A | 0,99531433 | 2561 |
| 2656 | A | G | 0,998367347 | 2457 |
| 2696 | A | G | 0,993723849 | 2393 |
| 2697 | C | T | 0,99164229 | 2393 |
| 2731 | G | A | 0,998248687 | 2290 |
| 2761 | A | G | 0,997774811 | 2248 |
| 2829 | A | G | 0,100458716 | 2181 |
| 2844 | C | A | 0,453707969 | 2176 |
| 2869 | G | A | 0,999061914 | 2135 |
| 2917 | C | T | 0,999085087 | 2189 |
| 2989 | A | G | 0,980811164 | 2293 |
| 3019 | C | T | 0,995938628 | 2216 |
| 3028 | C | T | 0,986561631 | 2161 |
| 3064 | T | C | 0,988832015 | 2149 |
| 3154 | T | A | 0,995386981 | 1952 |
| 3184 | A | G | 0,995945946 | 1480 |
| 3218 | C | T | 0,101419878 | 1479 |
| 3224 | T | G | 0,152159897 | 1552 |
| 3238 | T | C | 0,992287918 | 1559 |
| 3262 | G | A | 0,998729352 | 1577 |
| 3322 | C | T | 0,963707064 | 1543 |
| 3326 | A | G | 0,955174687 | 1541 |
| 3379 | C | T | 0,98853675 | 1483 |
| 3455 | T | A | 0,992625369 | 1356 |
| 3467 | G | T | 0,188118812 | 1314 |
| 3475 | C | T | 0,996120272 | 1031 |
| 3490 | T | C | 1 | 1009 |
| 3502 | C | T | 0,990816327 | 980 |
| 3541 | A | G | 0,999002991 | 1004 |
| 3546 | G | A | 0,989010989 | 1012 |
| 3571 | A | G | 1 | 959 |
| 3586 | G | A | 0,996808511 | 941 |
| 3613 | T | C | 0,995789474 | 950 |
| 3626 | G | A | 0,996852046 | 953 |
| 3638 | G | A | 1 | 943 |
| 3699 | T | C | 0,114649682 | 945 |
| 3715 | A | C | 0,997807018 | 915 |
| 3793 | A | G | 0,994541485 | 916 |
| 3802 | C | T | 0,990163934 | 918 |
| 3863 | T | A | 0,994394619 | 898 |
| 3913 | T | C | 0,997787611 | 904 |
| 3952 | T | C | 0,995735608 | 938 |
| 4003 | G | A | 0,998922414 | 928 |
| 4015 | G | T | 0,997844828 | 928 |
| 4039 | G | A | 0,995555556 | 900 |
| 4150 | G | A | 0,995485327 | 908 |
| 4273 | A | G | 0,988814318 | 894 |
| 4300 | T | C | 0,99113082 | 902 |
| 4474 | G | A | 0,997818975 | 923 |
| 4490 | T | C | 0,996791444 | 935 |
| 4549 | C | T | 0,986472425 | 961 |
| 4624 | A | G | 0,997830803 | 922 |
| 4669 | A | G | 0,997858672 | 938 |
| 4717 | A | G | 0,994594595 | 925 |
| 4765 | G | A | 0,996681416 | 904 |
| 4834 | C | T | 0,994375703 | 889 |
| 4849 | T | C | 0,989910314 | 892 |
| 4867 | T | C | 0,989714286 | 875 |
| 4891 | C | T | 0,992727273 | 827 |
| 4945 | T | C | 0,993614304 | 783 |
| 4969 | G | A | 0,99594046 | 739 |
| 5014 | C | T | 0,977941176 | 682 |
| 5089 | C | T | 0,991258741 | 573 |
| 5093 | G | A | 0,90199637 | 551 |
| 5116 | G | A | 0,94488189 | 508 |
| 5125 | T | C | 0,1133829 | 538 |
| 5149 | T | C | 0,993150685 | 730 |
| 5154 | C | T | 0,994825356 | 777 |
| 5176 | G | T | 0,985714286 | 911 |
| 5191 | G | A | 1 | 978 |
| 5230 | C | T | 0,988084326 | 1091 |
| 5257 | C | T | 0,987809316 | 5496 |
| 5326 | C | T | 0,995965983 | 9173 |
| 5359 | A | G | 0,12684613 | 9547 |
| 5371 | G | A | 0,999263855 | 9518 |
| 5473 | T | C | 0,995203837 | 8341 |
| 5515 | A | G | 0,980274203 | 7148 |
| 5566 | T | A | 0,998335553 | 6096 |
| 5572 | A | G | 0,97651117 | 6088 |
| 5581 | T | C | 0,996033058 | 6052 |
| 5626 | T | C | 0,994021651 | 6203 |
| 5657 | C | T | 0,999281093 | 6329 |
| 5674 | C | T | 0,968877711 | 6370 |
| 5683 | C | T | 0,99423676 | 6422 |
| 5707 | A | G | 0,802381321 | 6468 |
| 5722 | T | C | 0,996621622 | 6512 |
| 5728 | C | T | 0,995719309 | 6541 |
| 5746 | C | T | 0,997099237 | 6557 |
| 5752 | C | T | 0,997572816 | 6597 |
| 5764 | A | G | 0,95781862 | 6655 |
| 5770 | A | G | 0,98892562 | 6695 |
| 5776 | C | T | 0,986733002 | 6633 |
| 5848 | G | A | 0,997611353 | 7121 |
| 5920 | C | T | 0,997246317 | 7293 |
| 5926 | T | C | 0,993830546 | 7294 |
| 5998 | C | T | 0,97812328 | 7268 |
| 6046 | C | T | 0,993656611 | 7100 |
| 6055 | A | G | 0,989570296 | 7191 |
| 6082 | T | G | 0,997748065 | 7109 |
| 6191 | T | C | 0,995765606 | 7322 |
| 6208 | C | T | 0,996567349 | 7286 |
| 6250 | G | A | 0,989540049 | 6983 |
| 6262 | A | G | 0,997691531 | 6931 |
| 6310 | C | T | 0,989842329 | 6600 |
| 6313 | A | C | 0,884044669 | 6609 |
| 6331 | A | G | 0,996933456 | 6527 |
| 6340 | G | A | 0,998611754 | 6564 |
| 6352 | C | T | 0,996147326 | 6494 |
| 6382 | T | C | 0,998568475 | 6294 |
| 6394 | G | A | 0,997481108 | 6358 |
| 6415 | G | A | 0,991117345 | 6436 |
| 6418 | C | T | 0,997674779 | 6455 |
| 6481 | A | T | 0,979609929 | 6778 |
| 6634 | C | T | 0,997336258 | 6408 |
| 6655 | T | C | 0,998901099 | 6381 |
| 6697 | T | C | 0,946806737 | 5997 |
| 6745 | T | G | 0,994917631 | 5706 |
| 6748 | T | C | 0,994734995 | 5700 |
| 6790 | A | G | 0,99722313 | 6280 |
| 6796 | C | T | 0,995635306 | 6188 |
| 6841 | C | T | 0,997941409 | 6398 |
| 6937 | C | T | 0,996150762 | 6235 |
| 6991 | T | C | 0,994071816 | 5924 |
| 7019 | A | G | 0,987654321 | 4716 |
| 7023 | G | A | 0,991165697 | 4641 |
| 7066 | A | G | 0,982278481 | 4345 |
| 7075 | C | T | 0,978609626 | 4004 |
| 7087 | G | A | 0,993191935 | 3829 |
| 7159 | C | T | 0,993839836 | 3897 |
| 7164 | A | G | 0,971767078 | 3967 |
| 7171 | A | G | 0,996325662 | 5173 |
| 7228 | T | C | 0,996889096 | 6433 |
| 7276 | G | A | 0,998303359 | 5910 |
